# Loss of KLF14 triggers centrosome amplification and tumorigenesis

**DOI:** 10.1038/ncomms9450

**Published:** 2015-10-06

**Authors:** Guangjian Fan, Lianhui Sun, Peipei Shan, Xianying Zhang, Jinliang Huan, Xiaohong Zhang, Dali Li, Tingting Wang, Tingting Wei, Xiaohong Zhang, Xiaoyang Gu, Liangfang Yao, Yang Xuan, Zhaoyuan Hou, Yongping Cui, Liu Cao, Xiaotao Li, Shengping Zhang, Chuangui Wang

**Affiliations:** 1 Shanghai Key Laboratory of Regulatory Biology, Institute of Biomedical Sciences, East China Normal University, Shanghai 200241, China; 2 Institute of Translational Medicine, Shanghai General Hospital, Shanghai Jiao Tong University School of Medicine, No. 650 Xinsongjiang Road, Songjiang District, Shanghai 201620, China; 3 Department of General Surgery, Shanghai Eighth People's Hospital, Shanghai 200235, China; 4 Department of Pathology and Cell Biology, USF Morsani College of Medicine, 12901 Bruce B. Downs Boulevard, Tampa, Florida 33612, USA; 5 Department of Biochemistry and Molecular Cell Biology, Shanghai Jiao Tong University School of Medicine, Shanghai 200025, China; 6 Key Laboratory of Cellular Physiology, Ministry of Education, Shanxi Medical University, Shanxi 030001, China; 7 Key Laboratory of Medical Cell Biology, College of Translational Medicine, China Medical University, Shenyang 110000, China

## Abstract

Centrosome amplification is frequent in cancer, but the underlying mechanisms remain unclear. Here we report that disruption of the Kruppel-like factor 14 (*KLF14*) gene in mice causes centrosome amplification, aneuploidy and spontaneous tumorigenesis. Molecularly, KLF14 functions as a transcriptional repressor of Plk4, a polo-like kinase whose overexpression induces centrosome overduplication. Transient knockdown of KLF14 is sufficient to induce Plk4-directed centrosome amplification. Clinically, KLF14 transcription is significantly downregulated, whereas Plk4 transcription is upregulated in multiple types of cancers, and there exists an inverse correlation between KLF14 and Plk4 protein expression in human breast and colon cancers. Moreover, KLF14 depletion promotes AOM/DSS-induced colon tumorigenesis. Our findings reveal that KLF14 reduction serves as a mechanism leading to centrosome amplification and tumorigenesis. On the other hand, forced expression of KLF14 leads to mitotic catastrophe. Collectively, our findings identify KLF14 as a tumour suppressor and highlight its potential as biomarker and therapeutic target for cancer.

## Abstract (web summary)

Centrosome amplification is common in cancer, but the mechanism is not clear. Here the authors uncover a role for Kruppel-like factor 14 (KLF14) as a transcriptional repressor of polo-like kinase 4 (PLK4); KLF14 depletion correlates with increased PLK4 in human samples and leads to centrosome amplification and tumorigenesis in mice.

## Graphical abstract

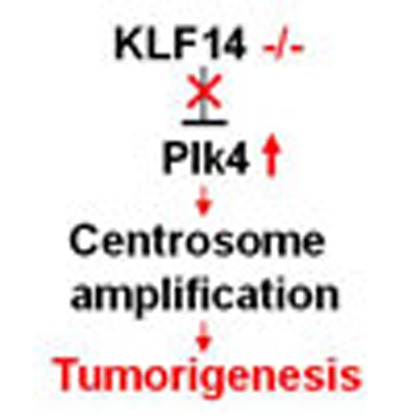

Centrosome amplification, the presence of extra centrosomes, is frequently detected in a growing list of human cancers, where it is proposed to play a causative role in tumorigenesis^1^,^2^. However, molecular mechanisms causing centrosome amplification in human cancers are still largely unknown^3^,^4^. Centrosome duplication is controlled by centriole replication. In most dividing animal cells, centrioles duplicate once and only once per cell cycle. Polo-like kinase 4 (Plk4 or SAK) has emerged as a master regulator of centriole assembly and duplication^5^,^6^. Depletion of Plk4 leads to a failure in the assembly of new centrioles and subsequent centrosome duplication defects, whereas, conversely, Plk4 overexpression induces extensive centrosome amplification^6^,^7^,^8^,^9^,^10^. Meanwhile, Plk4 heterozygous mice develop spontaneous liver and lung tumours^8^, whereas overexpression of Plk4 also promotes tumorigenesis in flies^11^,^12^. These studies prove that the protein level of Plk4 must be strictly regulated, to reduce the incidence of centrosome aberrations and tumorigenesis. Plk4 stability can be controlled by proteins including SCF/Slimb, Cullin 1, phosphatase 2A and SAPKKKs^7^,^13^,^14^,^15^,^16^,^17^, and its kinase activity also regulates its own stability^18^,^19^. However, regulation of Plk4 at the transcriptional level has not received much attention. In fact, protein levels of Plk4 mirror its transcripts, which are low in G1, and gradually increases through S and G2 to reach a maximum in mitosis, suggesting that gene transcription has a significant impact on controlling the overall Plk4 expression^20^,^21^. Therefore, identifying factors that govern Plk4 transcription would probably be rewarding in understanding the causes or effects of centrosome amplification in cancer.

The Krüppel-like family of transcription factors (KLFs) has received intensive investigations in human physiological and pathological processes^22^,^23^,^24^,^25^. In mammals, 17 KLF genes (*KLF1*–*KLF17*) have been identified, and both tumour suppressive and oncogenic functions have been defined for different KLFs^22^,^26^. In recent years, KLF14 (also called BTEB5) has elicited significant attention. Genetic studies have revealed that KLF14 may serve as a master regulator of gene expression in adipose tissue^27^, and there appears to be a connection between KLF14 and hypercholesterolemia and type 2 diabetes^28^. A recent report suggested that KLF14 functions as an activator for the generation of lipid signalling molecules^29^. These studies have provided useful information regarding KLF14 function in metabolism. In this study, we disrupted the *KLF14* gene in mice and report that KLF14 serves as a novel tumour suppressor essential for limiting Plk4-directed centrosome amplification.

## Results

### KLF14 deficiency leads to spontaneous tumorigenesis in mice

KLF14 has recently elicited attention as master regulator of lipid metabolism, but its physiological function is still unknown. Given there is growing realization that cancer is primarily a metabolic disease, we explored KLF14 transcript levels in human cancers using the Oncomine cancer profiling database. We observed that the expression of KLF14 messenger RNA is significantly reduced in many types of human cancers, including the breast, lymphatic, cervical, oral cavity, floor of mouth, pancreas and colorectal cancers, except for the Eckerle lymphoma data set (which shows a KLF14 mRNA upregulation pattern in the anaplastic lymphoma kinase-negative anaplastic large-cell lymphoma) (Supplementary Table 1).

**Figure 1:**
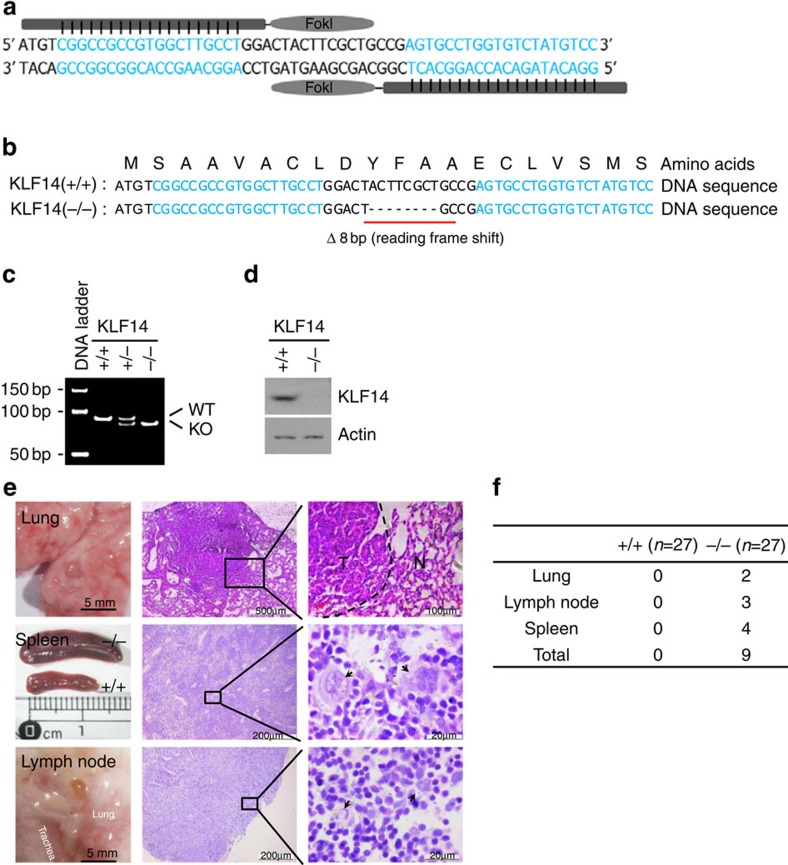
KLF14 KO mice develop spontaneous tumours. (**a**–**d**) Generation of KLF14 KO mice using TALENs. (**a**) Schematic drawing of TALENs and the recognition sequences of KLF14 (in blue). (**b**) KLF14 DNA and amino acid sequences from live WT (+/+) and KLF14-KO (−/−) mice. ‘− − −' denotes the eight nucleotides deleted. (**c**) PCR genotyping of WT and KLF14-KO mice. One upper band, WT (+/+); two bands, heterozygous (+/−); one lower band, KO (−/−). (**d**) MEFs from WT and KLF14-KO mice were analysed for KLF14 and actin (as protein loading control) expression levels by western blotting. (**e**,**f**) Loss of KLF14 induces spontaneous tumorigenesis. (**e**) Macroscopic view of tumour-bearing organs (left panel) and microscopic view of haematoxylin and eosin staining of tumour tissue sections (middle and right panels) in 13- to 14-month-old KLF14-KO mice. Right panel shows enlargements of boxed areas. Dotted line denotes border between normal (N) and tumour (T) tissue. Pathologic analysis revealed that KLF14-KO mice developed pulmonary adenomas and lymphomas in the spleen and lymph node. Splenomegaly was observed in mice with spleen tumours. Lymphoma of the spleen exhibits the effacement of lymphoid follicle and the appearance of typical neoplastic Reed/Sternberg cells (arrow). Lymphoma developed in lymph node exhibits the hallmark cells with horse-shoe-shaped nucleus (arrow). (**f**) Spontaneous tumour incidences in KLF14-WT (+/+) and -KO (−/−) mice. The frequency of spontaneous tumours in KO mice (9 of 27 in total) versus WT mice (0 of 27 in total) was statistically significant (*P*<0.01).

To determine the role of KLF14 in cancer, we generated KLF14 knockout (KO) mice using transcription activator-like effector nucleases^30^ (Fig. 1a). The intronless *KLF14* gene was disrupted by deleting eight nucleotides (confirmed by DNA sequencing), thus causing a frameshift mutation at the tenth amino acid and resulting in premature termination (Fig. 1b). The wild-type (WT, or +/+), heterozygous (+/−) and homozygous KO (−/−) mice were genotyped with PCR (Fig. 1c). Western blot analysis confirmed that there was no expression of KLF14 in mouse embryonic fibroblast (MEF) cells from KLF14-KO mice (Fig. 1d), indicating that the gene has been knocked out.

KLF14-KO mice were viable and showed no obvious abnormality in body weight and serum lipids (Supplementary Fig. 1), but developed spontaneous tumours over time. Starting from ∼11 months, the tumour incidence reached 33.3% at the age of 13∼14 months (Fig. 1e). Of 27 KLF14-KO mice studied, 2 (7.4%) developed lung adenomas, 4 (14.8%) developed lymphoma in the spleens and 3 (11.1%) developed lymphoma in the lymph nodes (Fig. 1f), whereas no spontaneous tumour was identified in other organs such as the heart, liver, kidney, breast, colon and thymus. The adenomas were relatively spherical lesions with discrete borders and composed of basophilic tumour cells uniform in appearance. The lymphomas were characterized by the Reed/Sternberg cells or large cells with horse-shoe-shaped nuclei surrounded by lightly staining eosinophilic area. As no tumour was detected in WT mice during this period, we conclude that loss of KLF14 leads to spontaneous tumorigenesis in adult mice.

### Loss of KLF14 causes genome instability

**Figure 2:**
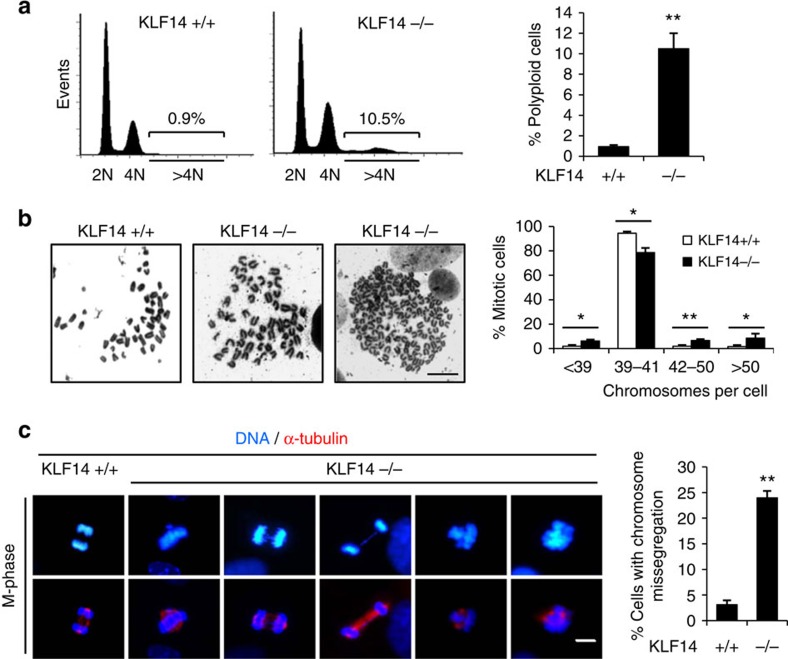
Loss of KLF14 induces genome instability. (**a**,**b**) KLF14 KO induces aneuploidy. (**a**) Cells with >4N DNA content increased in KLF14-KO MEFs. Representative flow cytometry profiles of KLF14-WT and -KO passage 3 (P3) MEFs stained with propidium iodide. Bar graph shows the mean percentage of cells with DNA content >4N. Data represent the mean±s.d. from three separate experiments, **P<0.01. (**b**) Representative images show Giemsa staining of metaphase chromosome spreads in KLF14-WT and -KO P3 MEFs (scale bar, 10 μm). Graph shows the fraction of MEFs with different numbers of chromosome per cell. One hundred metaphase spreads were counted for each group. Data represent the mean±s.d. from three separate experiments, *P<0.05, **P<0.01. (**c**) KLF14 KO induces chromosome instability. Spindles and DNA in KLF14-WT and -KO P3 MEFs were stained with α-tubulin (red) and 4,6-diamidino-2-phenylindole (DAPI, blue), and visualized by fluorescence microscopy. Representative images show mitotic chromosomal abnormalities including micronucleus, bridges, laggards and scattering in KLF14-KO MEFs (scale bar, 10 μm). Bar graph shows the percentages of MEFs with mitotic chromosomal abnormalities. More than 100 mitotic cells were counted for each group; data represent mean±s.d., **P<0.01.

To analyse the tumour suppressive activity of KLF14, we generated MEFs from 13.5-day-old embryos. MEFs at passage 3 were subjected to flow cytometry analysis. Polyploid cells with DNA content greater than tetraploid (>4N) were detected in KLF14-KO MEFs (Fig. 2a), indicating that loss of KLF14 induces polyploidy. Metaphase chromosome spread analysis confirmed that ∼20% of KLF14-KO MEFs were aneuploid (chromosome numbers ranging from 42 to 80 per cell), whereas <4% of WT MEFs were aneuploid (Fig. 2b). In addition, we detected >16% of mitotic KLF14-KO passage 3 MEF cells undergoing chromosome missegregation, whereas only ∼3% WT MEFs exhibited defective segregation (Fig. 2c). These results indicate that loss of KLF14 leads to chromosome instability *in vivo*.

### KLF14 reduction leads to centrosome amplification

**Figure 3:**
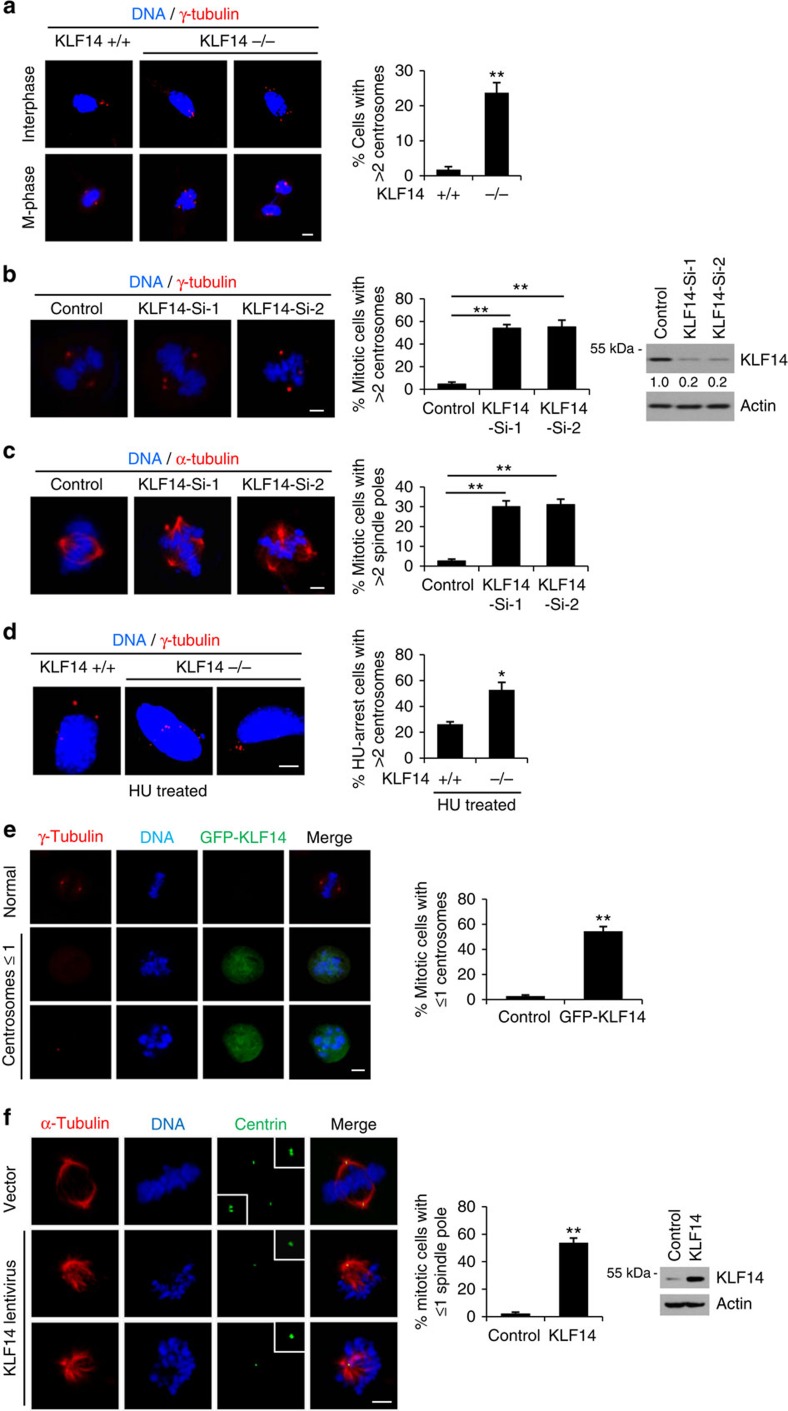
KLF14 regulates centrosome integrity and mitosis. (**a**–**d**) KLF14 reduction induces centrosome amplification. (**a**) Representative images show centrosome morphologies in KLF14-WT and -KO passage 3 (P3) MEFs (scale bar, 5 μm). Centrosomes and DNA were costained with anti-γ-tubulin antibody (red) and 4,6-diamidino-2-phenylindole (DAPI, blue), and visualized by fluorescence microscopy. Bar graphs show fraction of MEFs containing extra centrosomes. More than 100 cells per experimental group were counted; data represent mean±s.d., **P<0.01. (**b**,**c**) HeLa cells were infected with pLKO.1 vector (Control) or KLF14-knockdown (Si-1 or Si-2) lentivirus for 72 h. Centrosomes (**b**) and spindles (**c**) were stained with γ-tubulin (red) and α-tubulin (red), respectively (scale bar, 5 μm). DNA was stained with DAPI. Bar graphs show fraction of cells with indicated events. More than 100 cells per condition were counted from 3 independent experiments, **P<0.01. Representative western blotting showing KLF14 knockdown efficiency. (**d**) MEF cells from KLF14-WT and -KO mice were treated with 4 mM HU for 40 h and analysed for centrosome amplification. Centrosomes and DNA were stained and visualized as in **a** (scale bar, 5 μm). Bar graphs showing fraction of HU-treated cells containing extra centrosomes. More than 100 cells per condition were counted from 3 independent experiments, *P<0.05, **P<0.01. (**e**,**f**) KLF14 overexpression causes centrosome and spindle pole defects. (**e**) HeLa cells were transfected with vector (Control) or GFP-KLF14 plasmids for 48 h. Centrosomes and DNA were stained and visualized as in **a** (scale bar, 5 μm). Graphs show fraction of cells with aberrant centrosome in mitosis. More than 100 cells per experimental group were counted from 3 independent experiments, **P<0.01. (**f**) HeLa cells were infected with vector or KLF14 overexpression lentivirus for 48 h. Spindles poles, centrioles and DNA were costained with α-tubulin (red), centrin (green) and DAPI (blue), and visualized by fluorescence microscopy (scale bar, 5 μm). The boxed enlargements show centriole pairs in cells. Bar graphs show fraction of cells with aberrant spindle pole numbers in mitosis. More than 100 cells per experimental group were counted from 3 independent experiments, **P<0.01. Representative western blotting showing KLF14 expression.

Centrosome amplification has long been associated with genome instability and tumorigenesis^31^. We next examined centrosome integrity in KLF14-KO MEFs. As expected, KLF14 KO remarkably increased the incidence of centrosome amplification (>2 centrosomes per cell) in both interphase and mitotic MEFs (Fig. 3a). Consistently, we observed increased proportions of cells with centrosome amplification (Fig. 3b) and spindle multipolarity (Fig. 3c) in KLF14-knockdown HeLa cells. To determine whether KLF14 deficiency-induced centrosome amplification arises from failed cytokinesis, cells were exposed to S-phase arrest-inducing agent hydroxyurea (HU)^32^,^33^. We observed that, under long-term HU treatment, KLF14-KO MEF cells still displayed significantly increased frequency of centrosome amplification compared with the WT cells (Fig. 3d), suggesting that KLF14 deficiency causes centrosome amplification through a cytokinesis failure-independent manner. Moreover, we observed that KLF14 overexpression remarkably increased the percentage of mitotic cells with one or less focused centrosome and spindle pole, and the centrin at the monopolar spindle is often recognized as a single focus (Fig. 3e,f). Together, these results indicate that KLF14 plays a crucial role in keeping centrosome and spindle integrity.

### KLF14 is a transcriptional repressor of Plk4

**Figure 4:**
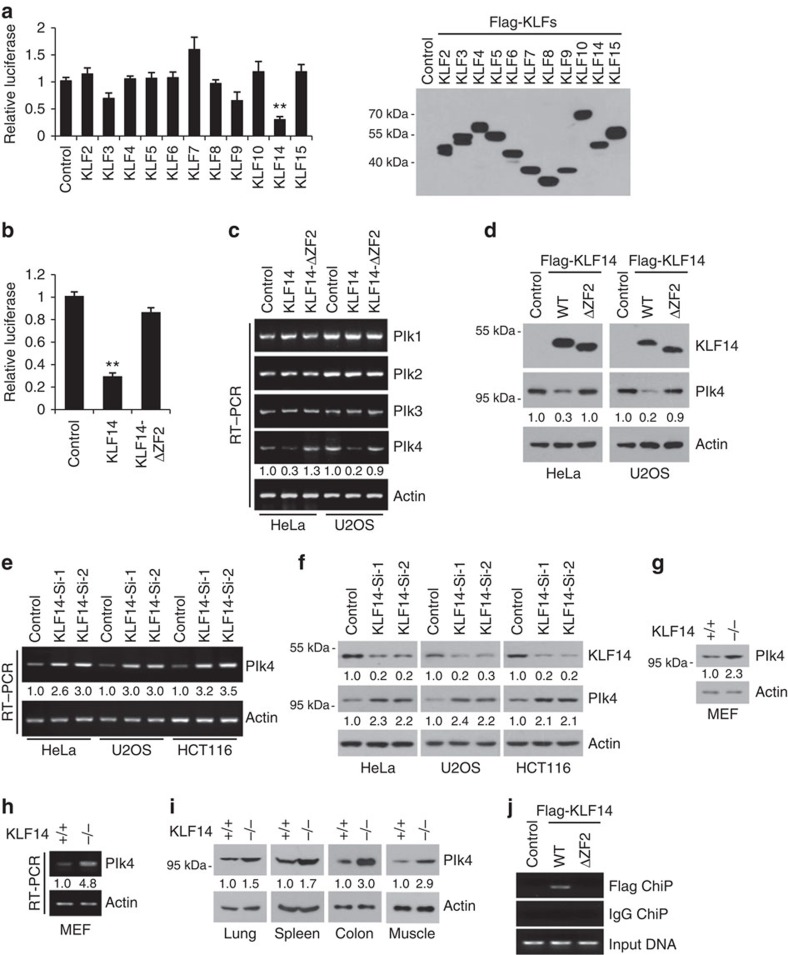
KLF14 inhibits Plk4 expression. (**a**–**d**) KLF14 overexpression inhibits Plk4 transcription and protein expression. (**a**,**b**) Human Plk4 promoter (−1343 to −141 bp upstream of ATG) luciferase reporter was co-transfected with indicated Flag-tagged KLFs (**a**) or KLF14-ΔZF2 (deletion of zinc finger 2) (**b**) in HeLa cells for 24 h, then relative luciferase activity was analysed. Data represent mean±s.d., **P<0.01. Representative western blotting showing the expression of the Flag-tagged KLFs detected with anti-Flag antibody. (**c**,**d**) HeLa and U2OS cells were transfected with Flag-KLF14 (WT) or Flag-KLF14-ΔZF2 (ΔZF2) for 36 h. The mRNA levels of Plks (Plk1-4) and β-actin (as internal standard) were analysed by reverse transcription–PCR (RT–PCR) (**c**). Protein levels of Flag-KLF14 and Plk4 were examined by western blotting using anti-Flag and anti-Plk4 antibodies, respectively (**d**). Relative levels of Plk4 mRNA and protein bands were quantified using densitometric analysis. (**e**,**f**) KLF14 knockdown increases Plk4 expression. HeLa, U2OS and HCT116 cells were infected with pLKO.1 vector (Control) or KLF14-knockdown (Si-1 or Si-2) lentivirus for 72 h. The mRNA and protein levels of endogenous Plk4 and KLF14 were examined by RT–PCR (**e**) and western blotting (**f**). Relative levels of Plk4 mRNA and protein bands were quantified using densitometric analysis. (**g**–**i**) KLF14 KO increases Plk4 expression. (**g**,**h**) The protein and mRNA levels of Plk4 in WT and KLF14-KO passage 3 (P3) MEFs were examined by western blotting (**g**) and RT–PCR (**h**). Relative levels of Plk4 mRNA and protein bands were quantified using densitometric analysis. (**i**) Plk4 protein levels in indicated tissues of KLF14-WT and -KO littermate mice were examined by western blotting. (**j**) KLF14 binds to Plk4 promoter. HeLa cells transfected with vector (Control), Flag-KLF14 (WT) or Flag-KLF14-ΔZF2 (ΔZF2) were processed for chromatin immunoprecipitation using FLAG M2 beads. Co-precipitated chromatin DNA was analysed by PCR using a pair of primers that amplify the −463 to −318 bp region of Plk4 promoter.

The above results suggest that KLF14 depletion may cause centrosome amplification and thus promotes tumorigenesis. In mammalian cells, Plk1–4 play essential roles in cell division and checkpoint regulation of mitosis^34^,^35^. Plk4 is emerging as a master regulator of centriole duplication and its aberrant expression leads to centrosome aberrations and tumorigenesis^5^,^6^,^36^,^37^. Scanning DNA sequences for transcription factor binding using TRANSFAC database, we identified multiple potential binding sites for KLFs in Plk4 promoter region. Therefore, we examined whether KLFs regulate Plk4 transcription. Interestingly, among the examined KLFs, KLF14 showed a strong inhibitory effect on Plk4-promoter luciferase activity (Fig. 4a). Moreover, deletion of zinc finger 2 in KLF14 (ΔZF2) remarkably eliminated the inhibitory effect (Fig. 4b). Similar results were observed at both the mRNA and protein levels of Plk4 in KLF14/ΔZF2-transfected cells (Fig. 4c,d). In contrast, KLF14 showed no effect to Plk1–3 mRNA levels (Fig. 4c). Furthermore, knockdown of KLF14 remarkably increased Plk4 transcription and protein expression (Fig. 4e,f), and Plk4 expressions were also elevated in KLF14-KO MEFs and tissues (Fig. 4g–i). In addition, full-length KLF14 (but not ΔZF2) binds to Plk4 promoter (Fig. 4j). Using promoter deletion and bioinformatics analyses (TRANSFA Public), we identified five putative Sp/KLF-binding motifs within the −477/−141 bp Plk4 promoter region. Double mutation of two Sp/KLF-binding motifs in Plk4 promoter remarkably abolished KLF14-mediated repression (Supplementary Fig. 2). Taken together, these observations suggest that KLF14 functions as a transcriptional repressor of Plk4.

### KLF14 inhibits Plk4-directed centriole duplication

**Figure 5:**
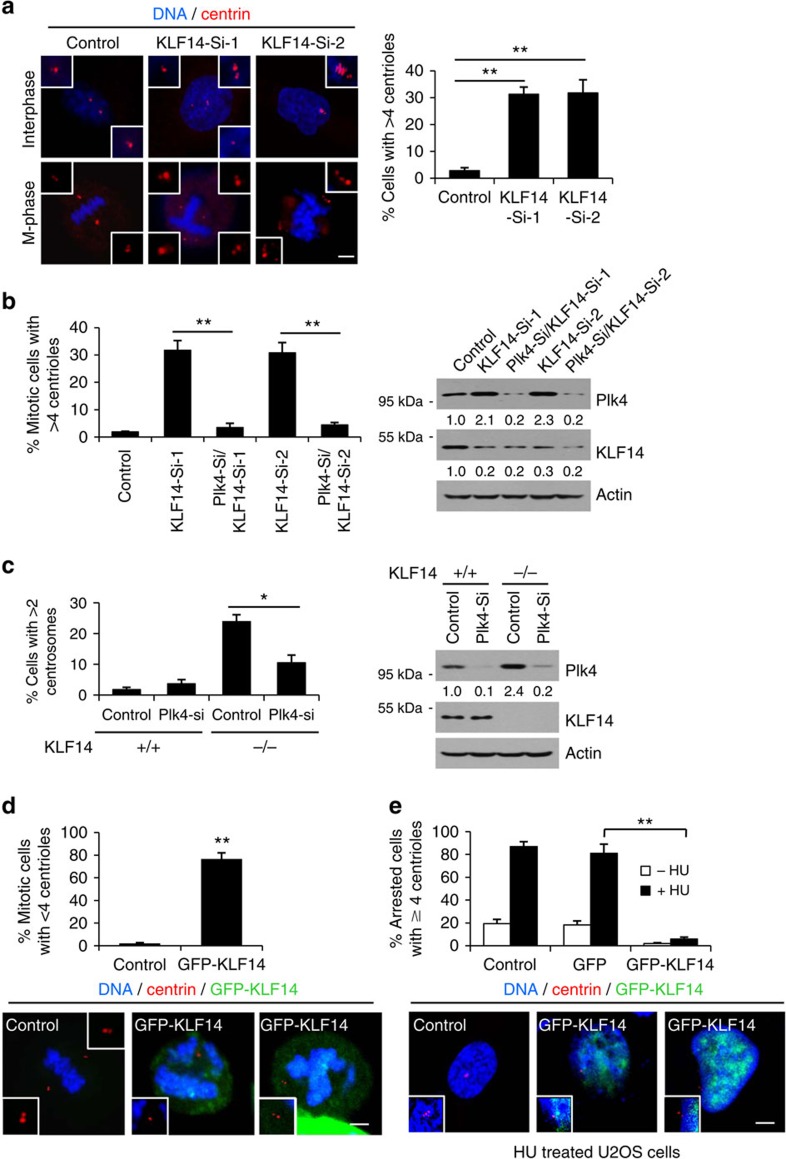
KLF14 inhibits Plk4-directed centriole replication. (**a**,**b**) KLF14 reduction induces centriole overduplication via Plk4 repression. (**a**) HeLa cells were infected with vector (Control) or KLF14-knockdown (Si-1 or Si-2) lentivirus for 72 h. Centrin (red) and DNA (blue) were costained with anti-centrin antibody and 4,6-diamidino-2-phenylindole (DAPI) and visualized by fluorescence microscopy (scale bar, 5 μm). Insets show magnification of the centriole area. Bar graphs showing fraction of cells with >4 centrioles per cell. More than 100 cells were counted from 3 independent experiments, **P<0.01. (**b**) HeLa cells were infected with indicated lentiviral constructs for 72 h. Centrioles were stained and visualized as in **a**. Bar graphs showing fraction of mitotic cells with >4 centrioles per cell. More than 50 cells per experimental group were counted from 3 independent experiments, **P<0.01. Representative western blotting showing knockdown efficiencies of KLF14 and Plk4. (**c**) KLF14-WT and -KO passage 3 (P3) MEFs were infected with vector (Control) or mouse Plk4-knockdown lentivirus for 48 h. Centrosomes and DNA were stained with γ-tubulin (red) and DAPI (blue). Bar graphs showing fraction of cells with >2 centrosomes per cell. Data represent mean±s.d. of three separate experiments, *P<0.05. Representative western blotting showing the knockdown efficiencies of Plk4. Relative protein levels of Plk4 were quantified by densitometry. (**d**,**e**) KLF14 overexpression inhibits centriole replication. (**d**) HeLa cells were transfected with vector (Control) or GFP-KLF14 for 48 h. Centrin (red) and DNA (blue) were stained and visualized as in **a** (scale bar, 5 μm). Insets show magnification of the centriole area. Bar graphs showing fraction of cells with aberrant centriole numbers in mitosis. More than 100 cells per experimental group were counted from 3 independent experiments, **P<0.01. (**e**) U2OS cells were transfected with green fluorescent protein (GFP) or GFP-KLF14 for 4 h followed by HU treatment for 48 h. Centriole (red) and DNA (blue) were stained and visualized as in **a** (scale bar, 5 μm). Insets show magnification of the centriole area. Bar graph showing percentage of cells with centriole overduplication during prolonged S-phase. More than 100 cells per experimental group were counted from 3 independent experiments, **P<0.01.

Next, we examined the extent to which Plk4 contributes to KLF14 deficiency-induced centrosome amplification. Results showed that knockdown of KLF14 remarkably increased proportions of cells with multiple centrioles (>4 per cell) (Fig. 5a), whereas double knockdown of KLF14 and Plk4 remarkably attenuated KLF14 reduction-induced formation of multiple centrioles (Fig. 5b). Consistently, Plk4 knockdown led to a significant reduction of the frequency of cells with extra centrosomes in KLF14-KO MEF cells (Fig. 5c). These data suggest that KLF14 reduction causes centrosome amplification through inducing Plk4-directed centriole overduplication. Conversely, KLF14 overexpression remarkably increased the percentage of mitotic cells with aberrant centriole numbers (<4 per cell) (Fig. 5d). To further confirm the inhibitory effect of KLF14 on centriole duplication, U2OS cells were treated with HU to induce multiple rounds of centriole duplication in the absence of DNA replication and mitotic division^32^. The number of centrin-labelled centrioles was counted in the GFP-KLF14-transfected cells. Remarkably, HU-induced centriole multiplication was significantly blocked by KLF14 overexpression (Fig. 5e). These results indicate that KLF14 plays a crucial role in repressing Plk4-directed centriole duplication.

### Negative correlation between KLF14 and Plk4 in human cancers

The above data strongly suggest that KLF14 may serve as a tumour suppressor via limiting Plk4-directed centrosome amplification. Indeed, the spontaneous tumours from KLF14-KO mice displayed high levels of Plk4 expression and centrosome amplification compared with WT mouse tissues (Supplementary Fig. 3). We therefore further examined the correlation between KLF14 and Plk4 in human cancers. Using Oncomine, we explored Plk4 transcription in human cancers listed in Supplementary Table 1. As shown in Supplementary Table 2, transcript levels of Plk4 in the breast, floor of mouth, lymphatic and colorectal cancers are clearly upregulated (redundant probe sets yield similar fold changes and significances). Plk4 transcription upregulation was also observed in the oral cavity, cervical and pancreatic cancers (at least one probe set shows a pattern of upregulation in each data set). Conversely, Plk4 transcription showed a downregulation pattern in the anaplastic lymphoma kinase-negative anaplastic large-cell lymphoma (Eckerle lymphoma data set), which showed a clear upregulation of KLF14 transcription. Taken together, results of Supplementary Tables 1 and 2 suggest a negative correlation between the transcript profiles of KLF14 and Plk4 in some types of human cancers.

**Figure 6:**
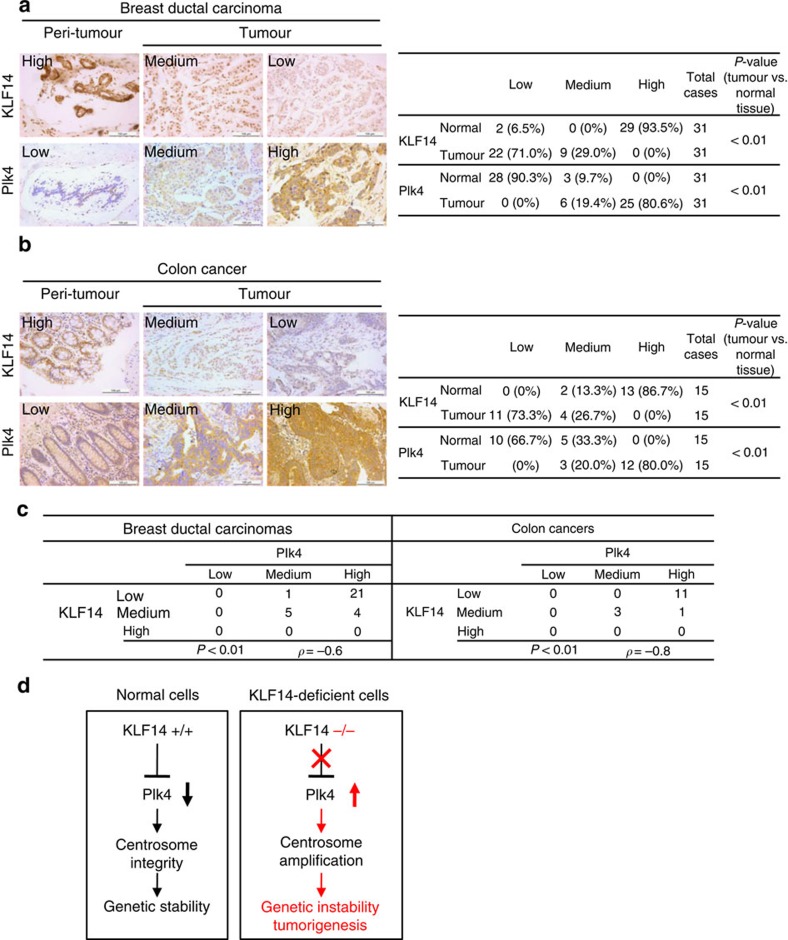
Immunohistochemical detection of KLF14 and Plk4 protein expressions in human cancers. (**a**,**b**) Comparison of KLF14 and Plk4 protein expression revealed by tissue array (breast cancer, 31 cases; colon cancer, 15 cases). Examples of immunohistochemical images of the adjacent normal tissue (peri-tumour) and tumour tissue in breast (**a**) and colon cancer (**b**) stained with anti-KLF14 and anti-Plk4 antibodies, respectively (scale bar, 100 μm). Levels of KLF14 and Plk4 expression were classified as high, medium and low according to the staining signals in each group (left panels). Tables show percentages of tissues with different levels of staining of KLF14 and Plk4 in normal and tumour tissues. (**c**) Spearman's *ρ*-coefficient test for evaluation of correlations between KLF14 and Plk4 immunohistochemical expression status in breast and colon cancer tissues. *r*<0 indicates negative correlation. The level of signification is expressed by the *P*-value. (**d**) A model illustrating role of KLF14 in maintaining genomic stability and suppressing tumorigenesis. KLF14 is required for limiting Plk4 transcription to maintain centrosome integrity in normal cells. Loss of KLF14 causes unlimited Plk4 expression, leading to centrosome amplification, genetic instability and thus tumorigenesis.

Given KLF14 transcription is significantly reduced, whereas Plk4 transcription is increased in a large number of human breast and colorectal cancers (Supplementary Tables 1 and 2), and a previous study also discovered Plk4 overexpression in human colon cancers^38^, we next performed immunohistochemistry staining of KLF14 and Plk4 protein on tissue chips containing paired tumour and peri-tumour normal specimens of human breast and colon cancers. Results showed that there were significantly lower protein levels of KLF14, but higher levels of Plk4, in cancer tissues of breast ductal carcinomas as compared with the adjacent normal tissues (Fig. 6a). Similar results were observed in human colon cancer samples (Fig. 6b). Correlational analysis revealed a significant negative correlation between the protein expression levels of KLF14 and Plk4 in both breast and colon tumour tissues (Spearman's *ρ*=−0.6 and −0.8, respectively) (Fig. 6c). As KLF14 inhibition promotes Plk4 transcription (Fig. 4), we suggest KLF14 reduction contributes to Plk4 overexpression in human cancers. Together, these results strongly suggest that there exists a negative correlation between Plk4 and KLF14 expressions in human cancers.

### Loss of KLF14 enhances AOM/DSS-induced colon tumorigenesis

The above data suggest that KLF14 reduction may increase the risk of breast cancer and colon cancer. However, no spontaneous breast and colon tumours were observed in KLF14-KO mice. To further address the role of KLF14 reduction in tumour formation, we used the azoxymethane (AOM) and dextran sulfate sodium (DSS) protocol^39^. Eight pairs of 8-week-old KLF14-WT and -KO mice were placed on the AOM/DSS protocol and the tumour formation was checked 6 weeks after the last DSS cycle (Supplementary Fig. 4a). As expected, AOM/DSS induction generated adenoma in the colon. Importantly, the KLF14-KO mice showed a marked increase in tumour number and size (Supplementary Fig. 4b–d), and the number of high-grade tumours was significantly higher in KLF14-KO mice (Supplementary Fig. 4e). These data suggest that KLF14 deficiency promotes tumour formation.

### KLF14 overexpression induces mitotic catastrophe

**Figure 7:**
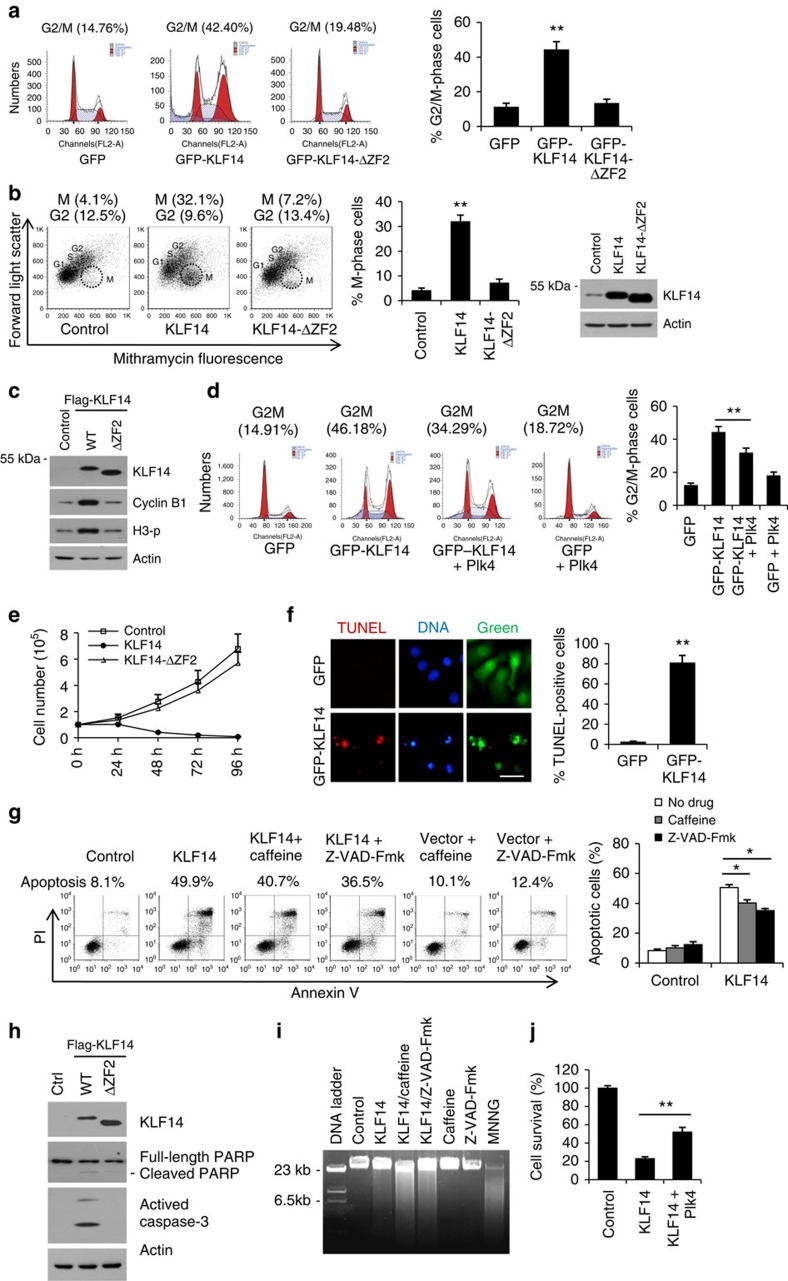
KLF14 overexpression induces mitotic catastrophe. (**a**) HeLa cells were transfected with green fluorescent protein (GFP), GFP-KLF14 or GFP-KLF14-ΔZF2 for 36 h. Cells were stained with propidium iodide (PI) and analysed by FACS. The DNA staining profiles of GFP-positive cells were displayed. Bar graphs showing per cent cells in G2/M-phase. (**b**) HeLa cells were infected with indicated lentiviral constructs for 36 h. Cells were stained with mithramycin A and analysed by FACS. Bargraphs showing per cent cells in M-phase. Western blotting showing KLF14 expression probed with anti-KLF14 antibody. (**c**) HeLa cells were transfected with indicated plasmids for 36 h and analysed for cyclin B1 and histone 3 phosphorylation (H3-p) levels by western blotting. (**d**) HeLa cells were co-transfected with indicated plasmids for 36 h, then stained with PI and analysed by FACS. The DNA staining profiles of GFP-positive cells were displayed. Bar graphs showing per cent cells in G2/M-phase. (**e**) 293T cells transfected with indicated plasmids were harvested at indicated time points and analysed for cell viability by MTT assay. (**f**) HeLa cells were transfected with GFP or GFP-KLF14 for 72 h and analysed by TUNEL assay (scale bar, 50 μm). Bar graph shows quantitative data of TUNEL-positive cells. More than 100 cells per experimental group were counted. (**g**) HeLa cells transfected with vector (Control) or KLF14 were treated with caffeine (2 mM) or caspase inhibitor Z-VAD-fmk (20 μM) for 60 h, then stained with Annexin V–fluorescein isothiocyante (FITC) and PI, and analysed by FACS. Bar graph shows percentage of apoptotic cells. (**h**) HeLa cells were transfected with indicated plasmids and analysed for activated caspase-3 and Poly (ADP-ribose) polymerase cleavage by western blotting. (**i**) HeLa cells transfected with vector (Control) or KLF14 were treated with caffeine or Z-VAD-Fmk for 60 h, and the DNA fragmentation was analysed by agarose gel electrophoresis. MNNG (500 μM, 4 h) serves as a positive control for necrosis. (**j**) Plk4 co-transfection decreased KLF14 overexpression-induced cell death. HeLa cells were transfected with indicated plasmids for 60 h and analysed for cell viability by MTT assay. All experiments were repeated three times, data represent mean±s.d., *P<0.05, **P<0.01. See also Supplementary Fig. 5.

Plk4 haploinsufficiency directly or indirectly leads to changes in the levels of RNA accumulation for a number of key cellular genes for several important cell cycle and DNA damage proteins^40^. Several studies suggest that Plk4 is required for proper cytokinesis and is essential for proper cellular proliferation and cell viability^6^,^8^,^10^,^40^,^41^. Recent reports showed that inhibition of Plk4 is a feasible strategy for cancer therapy^42^,^43^. We therefore further analysed whether transient induction of KLF14 regulates cell proliferation and survival. Fluorescenece-activated cell sorting (FACS) analysis revealed that KLF14 overexpression caused a threefold increase of cells in G2/M-phase, whereas KLF14-ΔZF2 had no such effects (Fig. 7a). We further distinguished G2 cells from cells in M-phase by flow cytometry analysis with mithramycin staining^44^ and revealed that overexpression of KLF14 but not KLF14-ΔZF2 leads to an increase of the proportion of cells in M-phase (Fig. 7b). Moreover, overexpression of KLF14, but not KLF14-ΔZF2, remarkably increased the levels of cyclin B1 and phosphor-histone H3 (two mitosis markers) (Fig. 7c). These data strongly suggest that enforced KLF14 expression causes mitotic arrest. Notably, we observed that coexpression of Plk4 only partially blocked KLF14 overexpression-induced cell cycle arrest at G2/M-phase (Fig. 7d), suggesting that mechanisms other than Plk4 inhibition also contribute to KLF14 overexpression-induced cell cycle arrest. In addition, we also observed that KLF14 overexpression remarkably increased signals of DNA damage marker γH2AX and phosphorylation levels of Chk2 at Thr68 and KAP1 at Ser824 (two downstream targets of ataxia telangiectasia mutated (ATM) in response to DNA damage) (Supplementary Fig. 5). Combining the above observations with the fact that transient induction of KLF14 causes aberrant centrosome and spindle pole numbers (Fig. 3e,f), we conclude that ectopic KLF14 expression results in abnormal mitotic features, increased DNA damage and mitotic arrest.

Deletion of centrosomal proteins can lead to aberrant spindle formation and subsequent mitotic catastrophe cell death^45^. A previous study reported that overexpression of a subset of KLFs, including KLF14, induces apoptotic cell death^46^. The fact that KLF14 regulates the mitotic machinery motivated us to further assess KLF14-induced cell death. Results showed that overexpression of KLF14, but not KLF14-ΔZF2, remarkably inhibited the growth of HeLa cells (Fig. 7e). Moreover, KLF14 overexpression remarkably increased the percentage of TUNEL (TdT-mediated dUTP nick end labelling)-positive cells (Fig. 7f) and the late apoptotic cells (Annexin-V positive and propidium iodide positive) (Fig. 7g). Meanwhile, the activated caspase-3 and the cleaved Poly (ADP-ribose) polymerase (PARP) were detected in KLF14, but not KLF14-ΔZF2 overexpression cells (Fig. 7h), indicating KLF14 overexpression induces cell death in a transcription-dependent manner. In recent years, the Nomenclature Committee on Cell Death defined mitotic catastrophe as one of the main forms of cell death that: (i) is initiated by perturbations of the mitotic apparatus; (ii) is initiated during the M-phase of cell cycle; (iii) is paralleled by some degree of mitotic arrest; and (iv) ultimately triggers cell death or senescence^47^. Given that KLF14 overexpression interferes with normal spindle formation, causes aberrant mitotic arrest and eventually leads to cell death, we conclude that KLF14 overexpression induces mitotic catastrophe.

Mitotic catastrophe is not a separate mode of cell death, but rather a process preceding apoptosis or necrosis^48^. Therefore, we tested whether the caspase inhibitor Z-VAD-Fmk (pan-caspase inhibitor) and caffeine (ATM/ataxia telangiectasia mutated/Rad 3-related protein (ATR) inhibitor) could block KLF14 overexpression-induced apoptosis. However, these two inhibitors only partially reduced KLF14-induced apoptosis (Fig. 7g). DNA fragmentation analysis showed that KLF14 overexpression resulted in accumulation of necrotic DNA fragmentation (large DNA fragment) (Fig. 7i). Moreover, both caffeine and caspase inhibitor treatments accelerated the accumulation of large DNA fragmentation, indicating that necrosis may play a leading role when the apoptosis was restrained in KLF14-overexpression cells. Notably, we observed that co-transfection of Plk4 partially reduced the cell death caused by KLF14 overexpression (Fig. 7j), indicating that KLF14 overexpression-induced cell death by mitotic catastrophe is associated with Plk4 reduction.

## Discussion

Centrosome amplification is a hallmark of cancer, but molecular mechanisms driving this phenotype remain unclear. In this study, we show KLF14 repress Plk4 transcription and transient knockdown of KLF14 is sufficient to induce Plk4-dependent centrosome amplification. Moreover, low KLF14 transcription is ubiquitously coupled with high Plk4 transcription in multiple forms of human cancers and there is a significant inverse correlation between the protein levels of KLF14 and Plk4 in human breast and colon cancers. These findings suggest that KLF14 reduction may cause unlimited Plk4-directed centrosome amplification and thus increase the risk of tumour formation. Indeed, many KLF14-KO mice developed spontaneous tumours and KLF14 depletion enhances AOM/DSS-induced colon tumour formation *in vivo*. On the other hand, we show that KLF14 overexpression induces mitotic catastrophe, suggesting a possibility for targeting KLF14 for cancer therapy.

In mammalian cells, centrioles organize the centrosome and accurate control of their numbers is essential for the maintenance of centrosome integrity. Plk4 emerges as a crucial regulator controlling centriole duplication. Currently, information regarding Plk4 regulation mainly focused on its degradation^21^. A recent study reported that E2F transcriptional factors (E2F1 and E2F3) positively regulate Plk4 transcription^49^. In this study, we show that KLF14 serves as a transcriptional repressor of Plk4 and there is a strong negative correlation between KLF14 and Plk4 expression *in vivo*. Consistent with the notion that loss of Plk4 prevents centriole formation, whereas its overexpression leads to *de novo* formation of centrioles^5^,^6^,^8^,^9^,^50^, we observed that KLF14 reduction induces formation of multipolar spindles with centriole overduplication, whereas depletion of Plk4 remarkably reduces KLF14-deficiency-induced centriole multiplication. These data indicate that KLF14 plays a vital role in Plk4 expression and centrosome integrity control.

Notably, stress-induced Plk4 activation induces chromosomal instability^17^ and Plk4 overexpression contributes to chromosome instability in gastric cancers^51^. In addition, Plk4 heterozygous MEFs showed a high incidence of chromosomal irregularities^52^ and a remarkable increase of mitotic cells was observed in Plk4-null mouse embryos^53^. These studies proved that any alterations of Plk4 expression or activity can cause chromosomal instability and mitotic errors. Consistent with our conclusion that KLF14 functions as a Plk4 transcription repressor, we show KLF14 depletion causes chromosomal missegregation and aneuploidy, whereas KLF14 overexpression causes abnormal mitotic features coupled with mitosis arrest. We conclude that strict control of KLF14 expression is required for chromosome stability and proper mitosis.

Mitotic catastrophe has been widely used to describe a form of cell death triggered by aberrant mitosis. Consistent with features of mitotic catastrophe^47^, our study shows that KLF14 overexpression leads to mitotic arrest with defective integrity of the mitotic spindle pole, DNA condensation, DNA fragmentation and finally cell death. Mitotic catastrophe usually drives cells to irreversible fates including apoptosis, necrosis or senescence^54^,^55^. Consistently, we observed that KLF14 overexpression induces both apoptosis and necrosis. Mitotic catastrophe has been considered as a process of cell death that results from dysregulated or failed mitosis^55^. Our results showed that co-transfection of Plk4 inhibited KLF14 overexpression-induced cell death, suggesting that Plk4 reduction, at least in part, contributes to KLF14-induced mitotic catastrophe. However, given Plk4 coexpression only partially blocked KLF14 overexpression-induced G2/M arrest and cell death, and KLF14 overexpression also induces a clear DNA damage response, we suggest that additional pathways may cooperate with the reduction of Plk4 in KLF14-overexpression cells to active mitotic catastrophe. Mitotic catastrophe acts as a mechanism to avoid genomic instability^54^,^56^. Our results show that KLF14 reduction causes genomic instability, whereas KLF14 overexpression induces mitotic catastrophe, suggesting that correct maintenance of KLF14 expression or activity under normal conditions is required for cells to keep their genome integrity, whereas under stress conditions cells may overactivate KLF14 to induce mitotic catastrophe, to avoid genomic instability. Further study on KLF14 regulation may provide novel insight for the prevention of genome instability.

Accumulating evidences suggest that centrosome amplification contributes to chromosome instability and clear evidence suggests that chromosome instability can cause tumour formation; however, whether centrosome dysfunction is a bystander or a driver of tumorigenesis is still a long-standing question^3^,^4^. According to previous reports, Plk4 is downregulated in hepatocellular carcinoma^57^, whereas upregulated in colon^38^ and gastric cancers^51^. In addition, increased rate of liver and lung cancers was detected in Plk4+/− mice^8^. Our results show that KLF14 is a transcriptional repressor for Plk4 and reduction of KLF14 causes centrosome amplification in a Plk4-dependent manner. Oncomine data analysis revealed that multiple human cancers are coincident with increased Plk4 transcription and decreased KLF14 transcription. Further analysis of human breast and colon cancer samples reveal that KLF14 protein expression is significantly decreased, whereas Plk4 expression is significantly increased, and a strong negative association exists between Plk4 and KLF14 expressions in the tumour tissues. Combining these findings with the facts that KLF14 KO causes spontaneous tumorigenesis in multiple organs, tumours from KLF14-KO mice exhibit amplified centrosomes with Plk4 upregulation and KLF14 deficiency promotes AOM/DSS-induced colon tumour formation *in vivo*, we suggest that KLF14 reduction can promote Plk4-directed centrosome amplification and consequently increase risks of tumour formation. However, our study does not exclude additional mechanisms mediating KLF14 deficiency-induced centrosome amplification and tumorigenesis. Clinically, centrosome amplification is frequently associated with tumour malignant transformation, progression, chemoresistance and patient prognosis^1^. Future clinical investigation of the dysfunction of KLF14 and its connection to centrosome amplification, genomic instability, cancer stage and prognosis may provide more valuable information regarding tumorigenesis and progression, and possibly new therapeutic strategy.

In conclusion, we indentify a function of KLF14 in maintaining the integrity of centrosome by limiting Plk4 transcription. Loss of KLF14 leads to centrosome amplification, genome instability and most importantly spontaneous tumour formation (Fig. 6d). Moreover, KLF14 is frequently downregulated in human cancers. Furthermore, enforced expression of KLF14 induces mitotic catastrophe. These findings reveal a tumour suppressor function of KLF14, highlighting its role as a potential biomarker and therapeutic target for cancer.

## Methods

### Generation of KLF14 KO mice

Target sites were ensured using the online software (http://boglabx.plp.iastate.edu/TALENT/help.php). TALE repeat domains and TALE nuclease expression vectors were generated as described by Qiu *et al*.^30^ TALEN expression vectors were linearized by NotI, purified by DNA clear-up kit and then transcribed using sp6 mMESSAGE kit (Ambion) following the manufacturer's instructions. Zygotes derived from superovulation of C57BL-6J females mating with the males were cultured in KSOM embryo culture medium. mRNA (40 ng μl^−1^) of each TALEN pair was injected into the cytoplasm of one-cell-stage embryos using an Eppendorf transferMan NK2 micromanipulator. Injected zygotes were transferred into pseudopregnant ICR female mice. The extracted DNA from newborn mice was amplified via PCR and then sequenced to find appropriate deficiency on *KLF14* genes. PCR analysis was performed to distinguish mice genotypes using 5′- CGCCGTGGCTTGC CTGGAC -3′ and 5′- TTCGGGGTCCGTTGCGCG -3′ primers. Mice with eight-nucleotides-deleted KLF14 (open reading disrupted) genotype were selected for breeding and crossed with C57BL-6J genetic background to produce F1 heterozygous offspring. All animals used in the studies were from F2 generations, which were derived from the intercross of F1 heterozygous offspring. Mice were housed in a specific pathogen-free facility on a 12 h light–dark cycle with *ad libitum* access to food and water. Animal use and care protocols, including all operation procedures, were approved by the Institutional Animal Care and Use Committee of East China Normal University.

### Western blotting

Cell lysate preparation and western blotting were performed as previously described^58^.

### Cell culture and materials

HeLa, U2OS, 293T, H1299, HCT116, KLF14-WT and -KO MEF cells were maintained in DMEM with 10% fetal bovine serum. KLF2-10, KLF14-15 and KLF14 deletion mutants and Plk4 coding sequences were cloned into pCDNA 3.0 vector or pLVX-IRES (lentiviral expression vector) by standard cloning methods. The pLKO.1-shRNAs targeting KLF14 and Plk4 double-stranded oligo nucleotides are 5′- CCGGTCATCCAGATATGATCGAGTACTCGAGTACTCGATCATATCTGGATGATTTTTG -3′ (KLF14-Si-1), 5′- CCGGGCTGCACCAAAGCCTATTACACTCGAGTGTAATAGG CTTTGGTGCAGCTTTTTG -3′ (KLF14-Si-2), 5′- CCGGGACCTTATTCACCAGTTACTTCT CGAGAAGTAACTGGTGAATAAGGTCTTTTTG -3′ (human Plk4-Si) and 5′- CCGGCTACT CGGTAAAGGATCATTTCTCGAGAAATGATCCTTTACCGAGTAGTTTTTG -3′ (mouse Plk4-Si), respectively (target sequence underlined). Ataxia telangiectasia mutated inhibitor (caffeine), caspase inhibitor (Z-VAD-Fmk) and DNA damage reagent (MNNG) were obtained from Sigma. Antibodies used were KLF14 (SAB1304202, Sigma), Plk4 (12952-1-AP, Proteintech), cyclin B1 (Santa Cruz, sc-752), activated-caspase-3 (BS7004, Bioworld Technology), p-Histone H3 (BS4094, Bioworld Technology), p-Chk2 (BS4043, Bioworld Technology), Poly (ADP-ribose) polymerase (13371-1-AP, Proteintech), Chk2 (1C12, CST) and anti-γH2AX (ab26350, Abcam). Uncropped scans of typical blots using some of the antibodies are shown in Supplementary Fig. 6.

### Luciferase reporter assay

Plk4 promoter (−1343 to −141 bp upstream of ATG) was subcloned into PGL4.17. Using this plasmid as template and the appropriate upstream primers, additional 5′-promoter deletion constructs were made: −1058LUC, −767LUC and −477LUC. The mutant promoter constructs were generated from the −477LUC construct using the QuickChange Site-Directed Mutagenesis kit. Luciferase reporter assay was performed as previously described^59^.

### Immunohistochemical staining

Human breast and colon cancer multiple tissue chips containing both tumour and the adjacent tissue sections (OD-CT-RpBre03-004 and HCol-Ade030PG-01) were purchased from Shanghai Outdo Biotech. The clinical characteristics of all of samples could be downloaded from the Web site: http://www.superchip.com.cn/web/index.asp. Antibodies against KLF14 and Plk4 were used for immunohistochemistry staining, respectively. The intensity of KLF14 and Plk4 staining was quantified as described previously^60^,^61^, scored and graded (low, 0–4 point; medium, 4–8 point; high, 9–12 point), respectively. To ensure an unbiased result, data were collected in a double-blinded manner. To analyse centrosomes in tissues, paraffin-embedded sections were heated for 10 min in a solution containing 0.2 mM EDTA, to render centrosome antigens before immunohistochemical staining with γ-tubulin.

### Colon carcinogenesis induction

Mice were administered with a single intraperitoneal injection of the AOM (10 mg per kg body weight) followed by 3 rounds of 2% DSS intake (5 days each round and 16 days interval between the 2 rounds). At the end of the AOM/DSS protocol, sections of tumours formed in distal and medial regions of colons from KLF14-WT and -KO mice were stained with standard haematoxylin and eosin and analysed by fluorescence microscopy.

### Aneuploidy analysis

Cells were treated with colcemid (50 ng ml^−1^, 37 °C, 6 h), collected, washed with PBS, suspended in KCl (75 mM, 37 °C, 15 min), fixed in Carnoy's solution for 30 min, then dropped onto slides and stained with 5% Giemsa solution. The chromosome number and morphology were analysed using confocal microscopy.

### Immunofluorescence staining

Immunofluorescence was performed as previously described^59^. Antibodies were purchased from Sigma (α-tubulin, γ-tubulin, centrin and Flag), Abcam (γH2AX) and Invitrogen (Alexa Fluor 568-conjugated IgG secondary antibody). Of note, cells were incubated at 4 °C for 1 h before permeabilization and fixation for centriole staining^9^.

### Cell cycle and cell death analyses

FACS and apoptosis assay were performed as previously described^59^. G2 and mitotic population were separated by staining with mithramycin A as previously described^44^. Briefly, cells were incubated with 0.1% NP40 in PBS for 5 min, followed by fixation with the addition of 4% formaldehyde in PBS at 4 °C for 15 min. DNA was stained with 20 μg ml^−1^ mithramycin A (Abcam) and analysed by FACS. Cell death was measured by MTT (3-(4,5-dimethylthiazol-2-yl)-2,5-diphenyltetrazolium bromide) and TUNEL assays. To analyse necrosis-associated DNA degradation, cells were lysed with lysis buffer (20 mM EDTA, 100 mM Tris, 0.8% SDS, pH 8.0) followed by RNase A (50 μg ml^−1^, 37 °C, 1 h) and Proteinase K treatment (100 μg ml^−1^, 50 °C, 5 h). The samples were separated on 0.5% agarose gel.

### Reverse transcription–PCR and chromatin immunoprecipitation

Reverse transcription–PCR analysis and chromatin immunoprecipitation were performed as previously described^62^. The following PCR primers were used: Plk1 (5′- CCAGCACGTCGTAGGATTCC -3′ and 5′- CAGTGGGATCTGTCTGAAGCAT -3′), Plk2 (5′- AAGGTGTTGACAGAGCCAGAA -3′ and 5′- GTCTGATTCACAGCCATGTCC -3′), Plk3 (5′- TGAGGACGCTGACAACATCTAC -3′ and 5′- GCGTGTAGTGAACCTGCTTGAT -3′), Plk4 (5′- ACTCACCCACAGACAACAATGC -3′ and 5′- CAACCAACGGAGATGTAATGCT -3′) and β-actin (5′- TCCTGTGGCATCCACGAA -3′ and 5′- TCGTCATACTCCTGCTTGC -3′). KLF14 chromatin immunoprecipitation was performed using Flag-M2 beads and the coprecipitated chromatin was analysed by PCR for the presence of Plk4 promoter DNA using 5′- TCAGCCATAAGTGTCCCATC -3′ and 5′- CCCAGGGTTCTAGACTTCG -3′ primers.

### Lipid analysis

Serum triglyceride, free fatty acid and cholesterol levels in KLF14-KO and WT mice were determined using Triglyceride Reagent (Sigma), NEFA-C Reagent (Wako) and Cholesterol Liquid Stable Reagent (Thermo), respectively. All assays were performed according to the manufacturer's instructions.

### Statistical analyses

Student's two-tailed *t*-test and GraphPad Prism were used for statistical analysis. Spearman's rank correlation test was used for correlational analysis. Differences were considered significant with a *P*-value<0.05.

## Additional information

**How to cite this article:** Fan, G. *et al*. Loss of KLF14 triggers centrosome amplification and tumorigenesis. *Nat. Commun.* 6:8450 doi: 10.1038/ncomms9450 (2015).

## Supplementary Material

Supplementary Information Supplementary Figures 1-6 and Supplementary Tables 1-2
