# Supplementary material for: Loss of KLF14 triggers centrosome amplification and tumorigenesis
Source: Nat Commun. 2015 Oct 6;6:8450. doi: 10.1038/ncomms9450 (PMC4600754; doi:10.1038/ncomms9450)
Supplement: Supplementary Information — Supplementary Figures 1-6 and Supplementary Tables 1-2 [file ncomms9450-s1.pdf]

Supplementary Figure 1

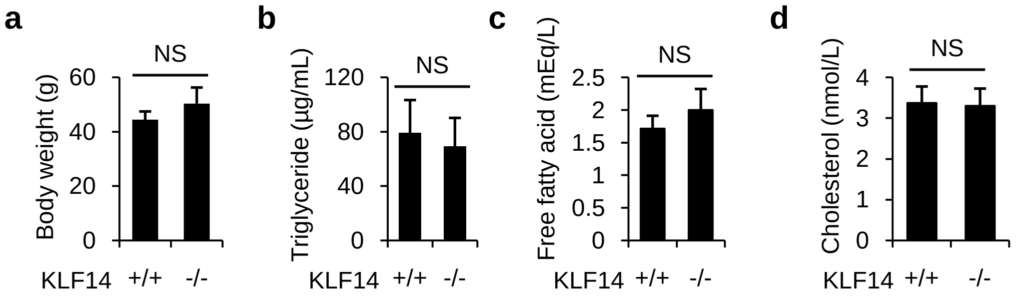

Supplementary Figure 1. KLF14 KO mice did not show any significant changes in body weight and serum levels of triglycerides, free fatty acids and cholesterol. Body weights (a) and levels of serum triglycerides (b), free fatty acids (c), and total cholesterol (d) of ~13-month-old KLF14 knockout (-/-) and wildtype (+/+) mice were determined. Data represent mean ± s.d., (n = 4-8).

Supplementary Figure 2

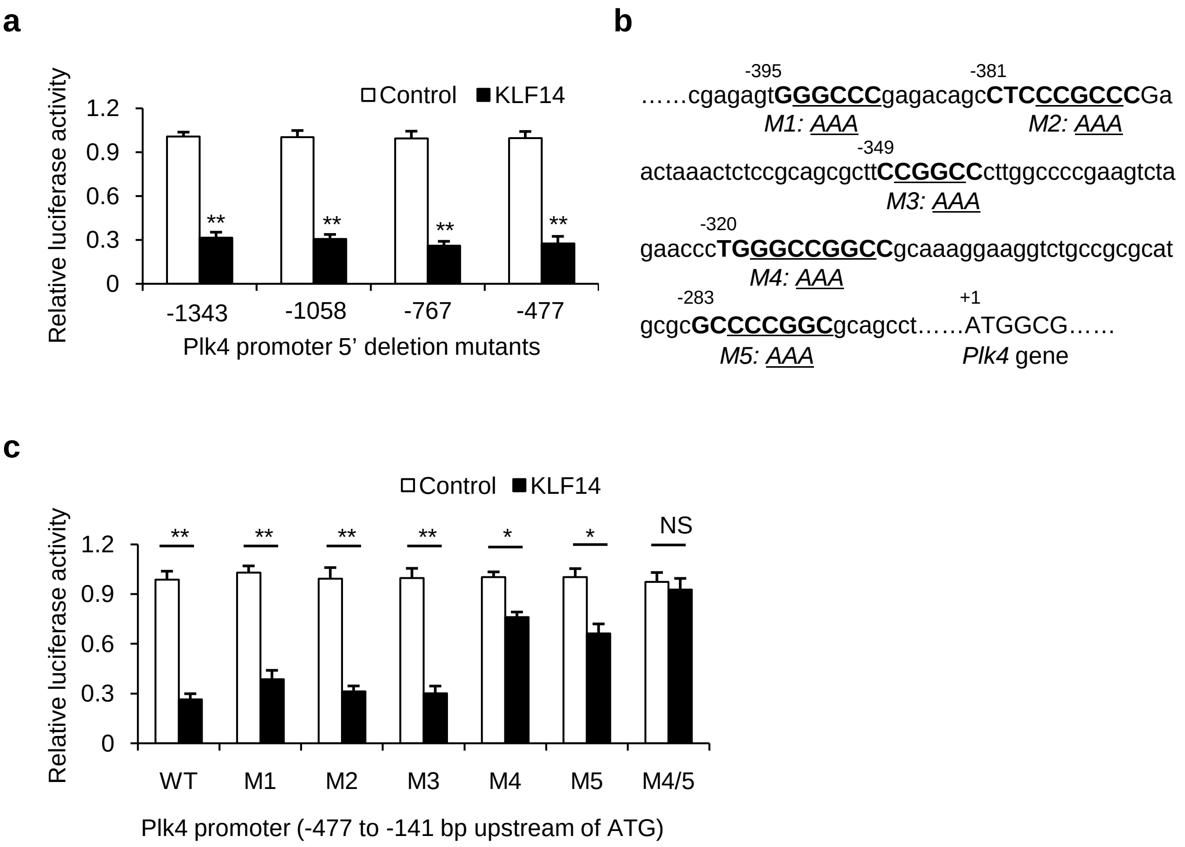

Supplementary Figure 2. Identification of KLF14 responsive site in human Plk4 promoter. (a) Deletion mutants of human Plk4 promoter constructs (-1343, -1058, -767 and -477 to -141 bp upstream of ATG) were cotransfected with KLF14 in HeLa cells for 24 h and analyzed for luciferase expression. Deletion of sequence upstream of -477 bp did not abrogate KLF14 response, suggesting a major KLF14 responsive element is located within the -477 downstream promoter region. Data represent the mean ± s.d. from three independent experiments, **p < 0.01. (b) Schematics showing the five putative Sp/KLF binding sites (indicated by capital letters) within the GC-rich region of the Plk4 promoter and the mutated derivatives (italic letters under the sequence). (c) Double point mutations were introduced in the -477 to -141 Plk4 promoter and tested for response to KLF14 in luciferase assay. Luciferase activities were normalized to the relevant controls. Data represent mean ± s.d., *p < 0.05, **p < 0.01, NS = nonsignificant.

Supplementary Figure 3

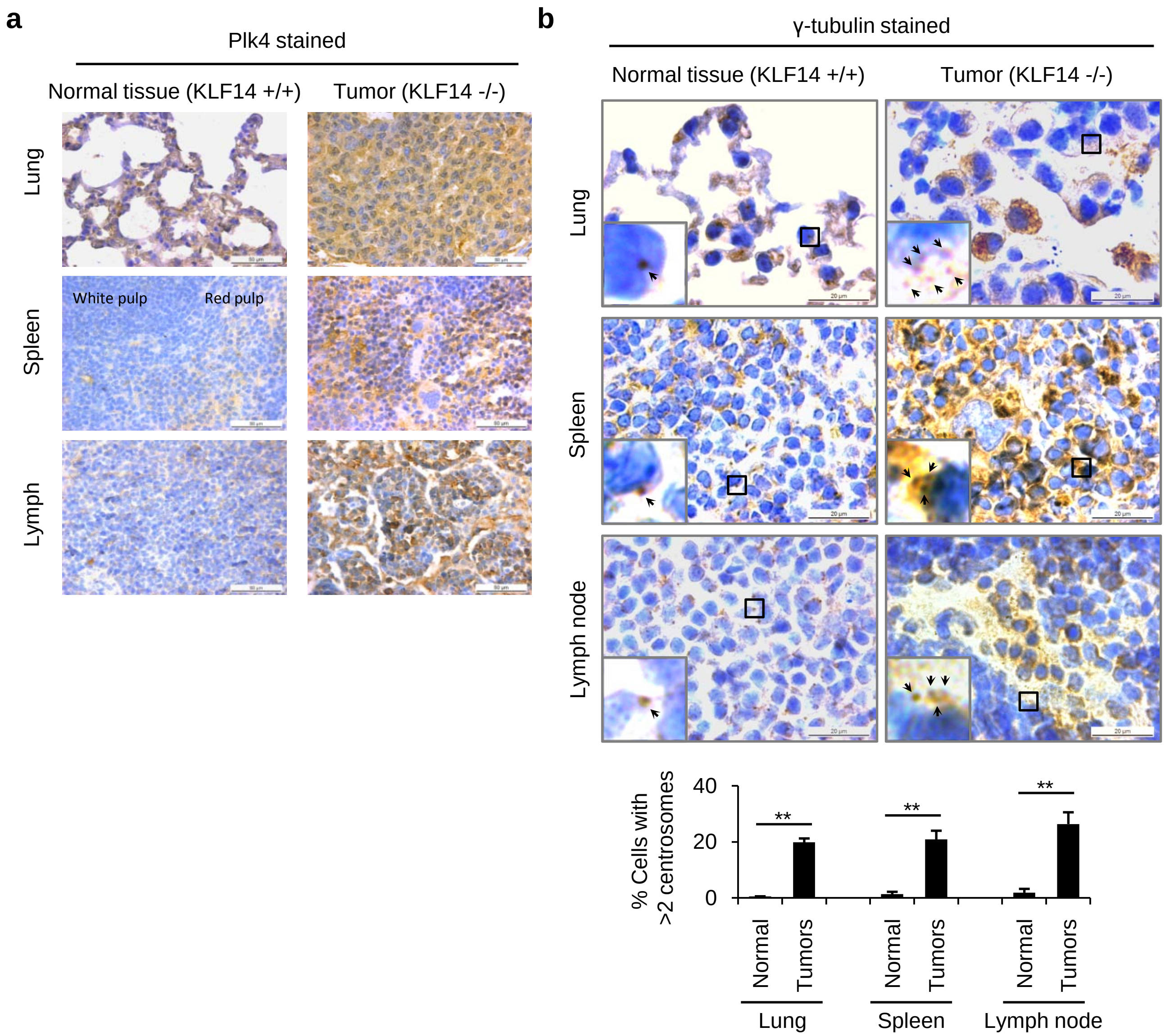

Supplementary Figure 3. Immunohistochemical staining for Plk4 and centrosomes in tumors and normal tissues derived from 13- to 14-month-old normal versus KLF14-KO mice (n = 2-3 for each group). Nuclei were counter-stained with DAPI. (a) Representative IHC images of anti-Plk4 stain (scale bar 50 μm). (b) Representative IHC images of anti-γ-tubulin stain (scale bar 20 μm). Insets show high magnification image of boxed areas (arrows point to the centrosomes). Bar graph shows the percent of cells with extra centrosomes. > 600 cells per experimental group were counted, data represent mean ± s.d., ** $p < 0.01$.

Supplementary Figure 4

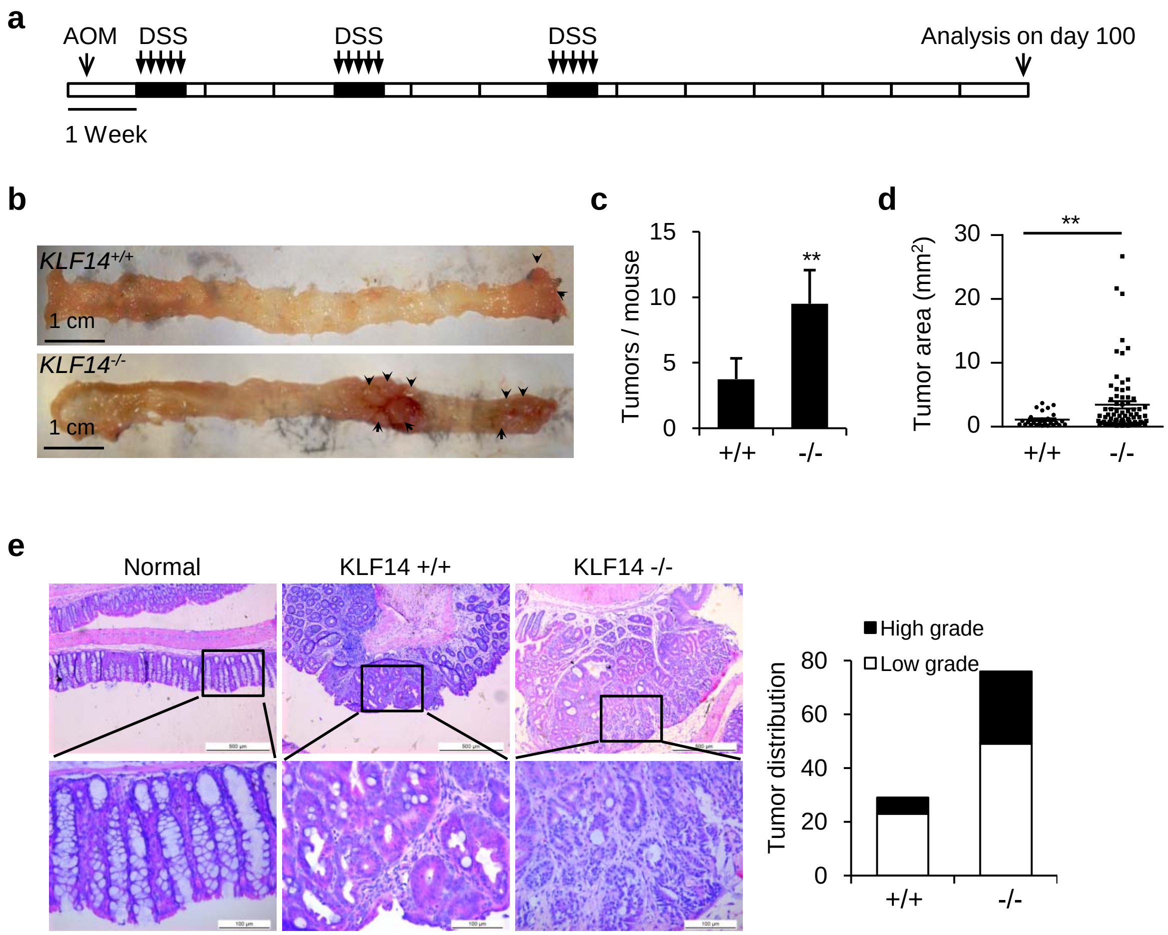

Supplementary Figure 4. KLF14 KO increases AOM/DSS-induced colon tumorigenesis. (a) Schematic representation of the AOM/DSS protocol. (b) Representative images showing colon tumor (arrowhead) formation. (c) Average number of tumors per mouse. Data represent mean ± s.d., $^{**}p < 0.01$. (d) Scatter plots show the size distribution of all tumors ($^{**}p < 0.01$). (e) Representative HE staining of tumor morphology. Lower images show enlargements of boxed areas. Tumor histologic grade was microscopically analyzed and classified into low or high. Graph shows the histologic grades of all tumors formed in KLF14-WT and -KO mice.

Supplementary Figure 5

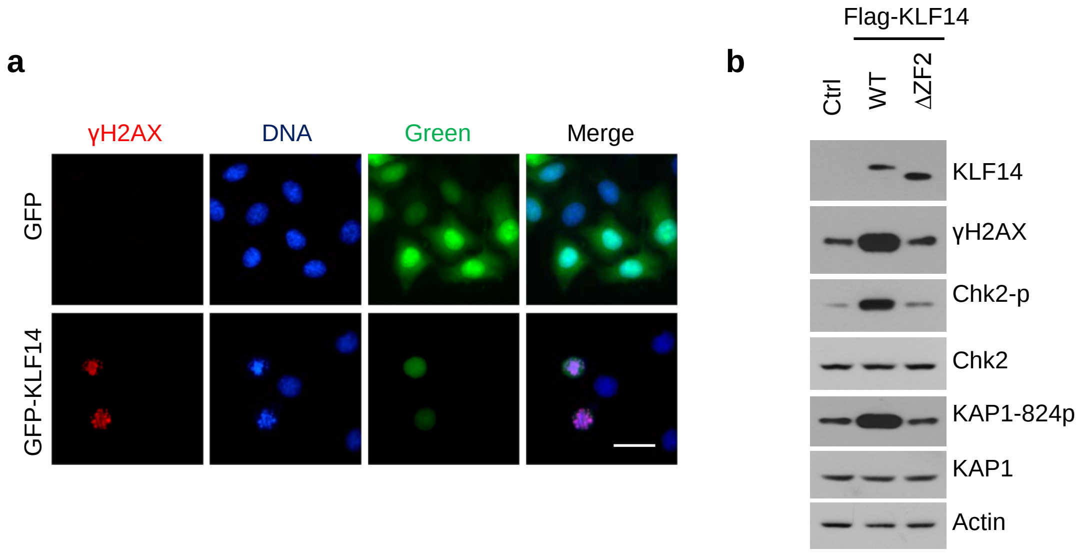

Supplementary Figure 5. KLF14 overexpression induces DNA damage. (a) γH2AX (red) in HeLa cells transfected with GFP-KLF14 for 36 h were stained and visualized by microscopy (scale bar 50μm). (b) HeLa cells were transfected with Flag-KLF14 (WT) and Flag-KLF14-ΔZF2 plasmids for 36 h and analyzed for levels of DNA damage markers (γH2AX, KAP1-Ser824 and Chk2-Thr68 phosphorylations) by western blot.

Supplementary Figure 6

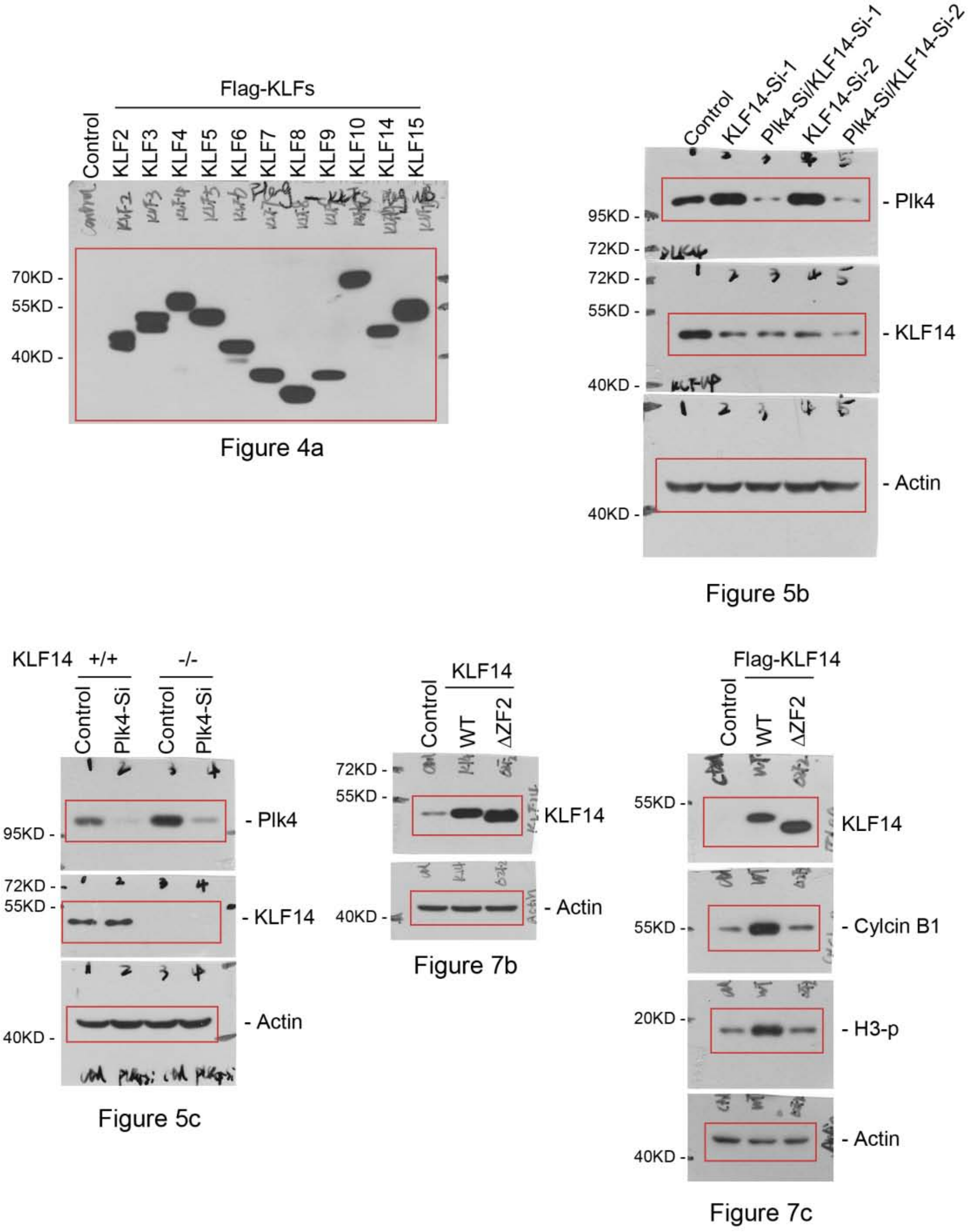

Supplementary Figure 6. Uncropped images of the original scans of representative immunoblots. Red boxes indicate the cropped areas displayed in the indicated figures.

Supplementary Table 1

Supplementary Table 1: KLF14 mRNA expression change in cancer versus normal tissues in the Oncomine database

Cancer type/subtype	KLF14 fold	P Value	Probe set	Database (Case Number)
Invasive Breast Carcinoma vs. Normal	-1.475	1.67E-06	A_23_P393626	TCGA Breast (593)
	-1.267	0.002	A_23_P393627	
Invasive Lobular Breast Carcinoma vs. Normal	-1.284	0.001	A_23_P393626	
	-1.147	0.05	A_23_P393627	
Angioimmunoblastic T-Cell Lymphoma vs. Normal	-1.192	2.26E-06	1552814_a_at	Piccaluga Lymphoma (60)
	-1.493	0.001	1552813_at	
Anaplastic Large Cell Lymphoma vs. Normal	-1.468	2.30E-05	1552813_at	
	-1.33	0.005	1552814_a_at	
Unspecified Peripheral T-Cell Lymphoma vs. Normal	-1.227	1.90E-05	1552814_a_at	
	-1.379	9.75E-05	1552813_at	
Cervical Cancer vs. Normal	-1.559	1.97E-07	1552813_at	Pyeon Multi-cancer (84)
	-1.277	3.54E-07	1552814_a_at	
Oral Cavity Carcinoma vs. Normal	-1.342	0.006	1552813_at	
	-1.231	0.007	1552814_a_at	
Floor of the Mouth Carcinoma vs. Normal	-1.274	0.002	1552813_at	
	-1.176	0.023	1552814_a_at	
Pancreatic Ductal Adenocarcinoma vs. Normal	-1.365	1.60E-08	1552813_at	Badea Pancreas (78)
	-1.428	2.53E-08	1552814_a_at	
Pancreatic Carcinoma vs. Normal	-1.291	0.012	1552814_a_at	Pei Pancreas (52)
	-1.165	0.022	1552813_at	
Cecum Adenocarcinoma vs. Normal	-1.246	0.007	A_23_P393626	TCGA Colorectal (237)
	-1.159	0.045	A_23_P393627	
Anaplastic Large Cell Lymphoma, ALK-Negative vs. Normal	1.312	0.003	1552813_at	Eckerle Lymphoma (64)
	1.202	0.046	1552814_a_at	

* Data were obtained from the cancer microarray database Oncomine (www.oncomine.org). The threshold search criteria: the expression of KLF14 gene was analyzed using at least two different probe sets, and the redundant probe sets showed similar over- and under-expression patterns (p-value <0.05 and the fold change ≥ 1.2, which is fairly low). A negative fold change indicates x-fold down-regulation of KLF14 gene expression in tumors compared with normal tissues, while a positive number indicates fold induction.

Supplementary Table 2

Supplementary Table 2**:** Plk4 mRNA expression change in cancer versus normal tissues in the Oncomine database

Cancer type/subtype	Plk4 fold	P Value	Probe set	Database (Case Number)
Invasive Breast Carcinoma vs. Normal	2.633	3.90E-21	A_23_P155968	TCGA Breast (593)
	2.586	4.38E-19	A_23_P155969	
	2.31	6.71E-20	A_23_P155971	
	1.512	1.15E-05	A_23_P912925	
Invasive Lobular Breast Carcinoma vs. Normal	1.913	8.45E-14	A_23_P155971	
	2.043	2.48E-12	A_23_P155968	
	1.995	1.73E-10	A_23_P155969	
	1.316	0.006	A_23_P912925	
Angioimmunoblastic T-Cell Lymphoma vs. Normal	2.291	2.34E-04	204886_at	Piccaluga Lymphoma (60)
	1.812	0.009	204887_s_at	
	1.536	0.031	211088_s_at	
Anaplastic Large Cell Lymphoma vs. Normal	2.521	5.95E-04	204886_at	
	2.257	0.002	204887_s_at	
	2.088	0.018	211088_s_at	
Unspecified Peripheral T-Cell Lymphoma vs. Normal	2.728	8.70E-09	204886_at	
	1.929	2.76E-07	204887_s_at	
	1.336	0.01	211088_s_at	
Cervical Cancer vs. Normal	2.881	1.06E-09	204887_s_at	Pyeon Multi-cancer (84)
	2.829	1.11E-09	204886_at	
	1.095	0.069	211088_s_at	
Oral Cavity Carcinoma vs. Normal	1.827	0.004	204886_at	
	1.672	0.06	204887_s_at	
	-1.04	0.66	211088_s_at	
Floor of the Mouth Carcinoma vs. Normal	2.959	1.46E-07	204887_s_at	
	2.454	9.67E-04	204886_at	
	1.135	9.00E-03	211088_s_at	
Pancreatic Ductal Adenocarcinoma vs. Normal	1.252	0.002	204887_s_at	Badea Pancreas (78)
	1.205	0.007	204886_at	
	1.033	0.188	211088_s_at	
Pancreatic Carcinoma vs. Normal	1.533	3.06E-05	204887_s_at	Pei Pancreas (52)
	1.079	0.218	204886_at	
	-1.069	0.059	211088_s_at	
Cecum Adenocarcinoma vs. Normal	2.184	4.88E-09	A_23_P155969	TCGA Colorectal (237)
	2.242	9.12E-09	A_23_P155968	
	2.01	2.07E-07	A_23_P155971	
	2.187	2.16E-07	A_23_P912925	
Anaplastic Large Cell Lymphoma, ALK-Negative vs. Normal	-1.31	3.09E-15	211088_s_at	Eckerle Lymphoma (64)

*Data were obtained from the cancer microarray database Oncomine (www.oncomine.org). A positive fold change indicates x-fold up-regulation of Plk4 gene expression in tumors compared with normal tissues, while a negative number indicates fold reduction.
